# The Severity of CVB3-Induced Myocarditis Can Be Improved by Blocking the Orchestration of NLRP3 and Th17 in Balb/c Mice

**DOI:** 10.1155/2021/5551578

**Published:** 2021-05-12

**Authors:** Jifei Chen, Fan Yang, Song Shi, Xuexiang Liu, Fengxian Qin, Xiaomou Wei, Yujie Huang, Wenwu Liang, Lin Miao

**Affiliations:** ^1^Medical Science Laboratory, The Fourth Affiliated Hospital of Guangxi Medical University, Liuzhou, Guangxi 545005, China; ^2^Internal Medicine-Cardiovascular Department, The Fourth Affiliated Hospital of Guangxi Medical University, Liuzhou, Guangxi 545005, China

## Abstract

**Background:**

The functional characteristics of NLRP3 in the pathogenesis of coxsackievirus B3- (CVB3-) induced viral myocarditis (VMC) have not been fully elucidated, and the targeted therapeutic effect of NLRP3 or its related pathway in VMC has not been reported.

**Method:**

In this work, the change patterns of NLRP3- and Th17-related factors were detected during the pathological process of CVB3-induced VMC in Balb/c mice. The correlation between NLRP3 and Th17 cells during the VMC process was analyzed by Spearman test. The coculture system of spleen CD4^+^ T and bone marrow CD11c^+^ DC cells was set to explore the orchestration of NLRP3 and Th17 in the pathological development of VMC in vitro. Anti-IL-1*β* antibody or NLRP3^−/−^ Balb/c were used to block the NLRP3 pathway indirectly and directly to analyze the NLRP3-targeting therapeutic value.

**Results:**

The change patterns of NLRP3- and Th17-related molecules in the whole pathological process of mouse CVB3-induced VMC were described. Through Spearman correlation analysis, it was confirmed that there was a close correlation between NLRP3 and Th17 cells in the whole pathological process of VMC. And the interaction mode between NLRP3 and Th17 was preliminarily explored in the cell experiment in vitro. Under the intervention of an anti-IL-1*β* antibody or NLRP3 knockout, the survival rate of the intervention group was significantly improved, the degree of myocardial inflammation and fibrosis was significantly alleviated, and the content of myocardial IL-17 and spleen Th17 was also significantly decreased.

**Conclusion:**

Our findings demonstrated a key role of the NLRP3 inflammasome and its close relationship with Th17 in the pathological progression of CVB3-induced VMC and suggested a possible positive feedback-like mutual regulation mechanism between the NLRP3 inflammasome and Th17 in vitro and in the early stage of CVB3 infection. Taking NLRP3 as a new starting point, it provides a new target and idea for the prevention and treatment of CVB3-induced VMC.

## 1. Introduction

According to the 2013 world census, the incidence of VMC (viral myocarditis) is about 22 per 100,000 people, and most of the patients are children or young adults [1]. In addition, it has been reported that 1/4 of dilated cardiomyopathy and myocarditis in children and young patients is caused by CVB3 (coxsackie virus group B type3) [2]. However, the pathogenesis of VMC has not been fully elucidated. And there is still no clinically proven effective treatment except supportive care [3]. While CVB3 replication could directly injure the myocardium, the excessive host immune response can also be activated by the recognition of CVB3 and NOD2 (nucleotide-binding oligomerization domain-containing protein 2) [4], which plays a critical pathogenic role during CVB3-induced myocarditis [5]. In addition, the overactivated immune response mediated by Th1, Th17, and Th2 is also an important cause of cardiomyocyte injury in VMC [6].

Th17 is a subset of T helper cells related to autoimmunity and inflammation. Our previous results showed that the proportion of Th17 cells in the spleen, the level of IL-17 mRNA in the ventricular muscle tissue, and the content of IL-17 protein in the serum were increased in VMC mice [7]. Besides, the application of the IL-17 antibody can partly reduce the injury of myocarditis caused by CVB3 [8]. It is suggested that Th17 subsets and their effector molecule IL-17 are closely related to the pathological process of VMC [6]. However, the interaction between Th17 and NLRP3 and its role in CVB3-induced myocarditis are rarely reported.

In CVB3-induced myocarditis, once CVB3 is recognized by NOD2, the NLRP3 (NOD-, LRR-, and pyrin domain-containing protein 3) inflammasome will be activated to transform pro-Caspase-1 precursors into active Caspase-1 through three modes of intracellular potassium efflux and lysosomal membrane permeability leading to the release of cathepsin B and ROS (reactive oxygen species). Then, IL-1*β* and IL-18 precursors are cut to form IL-1*β* and IL-18 mature bodies, respectively, which are released for exocytic function and trigger inflammatory reaction [4, 9, 10]. Recent studies have shown that CVB3 infection in vivo and in vitro can induce the activation of the NLRP3 inflammasome, and the activation of the inflammasome can promote CVB3-induced myocarditis. It is suggested that inflammasomes play an important role in viral myocarditis induced by CVB3 [5].

NLRP3 is a trisomy protein, which consists of an amino-terminal pyrin domain (PYD), a central NACHT domain, and a carboxy-terminal leucine-rich repeat domain (LRR domain), in which NACHT contains ATP activation sites. The NLRP3 inflammasome contains a sensor (NLRP3), an adapter (ASC), and an effector (Caspase-1). It is worth noting that NLRP3 is the only sensor that can be activated by various unrelated stimuli to sense cellular stress [4]. Increasing studies have shown that mutations or disorders of NLRP3 inflammasome components will lead to hereditary or acquired inflammatory diseases, including infectious, autoinflammatory, and autoimmune diseases [11–15]. Hence, the NLRP3 inflammasome and its related signal pathways provide a new idea for disease treatment strategies.

Up to now, it is exciting that a number of inhibitors that directly or indirectly inhibit inflammasome components or related pathways have been reported. In addition, inhibitors with high specificity and small molecules directly targeting NLRP3, such as MCC950, CY-09, oridonin, tranilast, MNS, and dapansutrile, have been reported in recent years [16–21]. Among them, MCC950 has been reported to be effective in a variety of preclinical immunopathological models, including cryopyrin-associated periodic syndrome, experimental autoimmune encephalomyelitis, Alzheimer disease, traumatic brain injury, atherosclerosis, cardiac arrhythmias, myocardial infarction, diabetes, steatohepatitis, and colitis [22–29]. However, in the treatment of viral myocarditis, direct or indirect inhibition of NLRP3 inflammasome components or related pathways has not been reported.

In this article, we would like to figure out whether the overactivated NLRP3 inflammasome is involved in the pathogenesis of CVB3-induced VMC or not, to disclose the correlation and interaction between NLRP3 inflammasome and Th17 cells in VMC process, and to explore the therapeutic value of NLRP3 inhibition in viral myocarditis by means of IL-1*β* antibody and NLRP3 knockout.

## 2. Materials and Methods

### 2.1. Animals and CVB3-Induced VMC Model

Wild type and NLRP3^−/−^ Balb/c were purchased from Beijing Vital River Laboratory Animal Technology Co., Ltd. CVB3 (Nancy strain) was activated and proliferated by a human laryngeal cancer cell line and harvested when the degree of cytopathic effect was more than 75%. TCID50 (median tissue culture infective dose) is 10^−7^. Each mouse in the VMC group was injected intraperitoneally with 0.1 ml saline containing the 100TCID50 virus, while the control group was injected with the same amount of saline [30]. The day of virus injection was set as 0 week, and at 0, 1, 2, 3, 4, and 6 weeks after injection, the mice were sacrificed and the heart and peripheral blood were collected. There were 10 mice in each group. The gRNAs used in NLRP3 knockout and gene identification primers and methods are listed below (Tables 1–3). The heart, spleen, and peripheral blood were collected from mice at the time of sacrifice for the next experiment.

### 2.2. Humane Endpoints

The health and behavior of experimental mice were monitored every day. The longest modeling lasted 6 weeks, and some of the experiments lasted 2 weeks. During the experiment, due to the characteristics of VMC disease, it will cause mouse shrugging, curling, hunchback, dull fur, reduced activity, irritability, and other symptoms. The basic principle was that the mice in each group were maintained to the end of the experiment as far as possible. Mice were sacrificed by cervical vertebrae dislocation after blood collection from the eyeball. If the mice in the group were in extreme pain, trembling, and other dying states, these mice would be euthanized and removed from the group. After injection of CVB3, the mice may attack each other. When this happens, the attackers will be transferred to other cages. The injury degree of other mice was evaluated, and the bites were handled properly.

### 2.3. Hematoxylin-Eosin (HE) Staining, Masson-Trichrome Staining, and Immunohistochemistry (IHC)

Hearts were cut longitudinally, fixed in 10% phosphate-buffered formalin, and embedded in paraffin. The myocardial tissues were cut into 4 *μ*m sections at various depths in the heart and stained with hematoxylin and eosin to determine the level of inflammation and graded in a blinded manner by three pathologists based on the following semiquantitative scale (Table 4) [31].

Masson-trichrome staining was performed according to the instruction of BASO (INS-MASS03) Masson stain procedure.

IHC was performed according to the instruction of the Abcam IHC procedure. Generally, 4 *μ*m thick paraffin sections were used in this study. After the section, half of the slides proceeded with HE stains and microscopic examination; the others were dewaxed in xylene and rehydrated in graded ethanol. Endogenous peroxidase activity was blocked by 0.3% H_2_O_2_ for 10 minutes. Antigen retrieval was carried out by heat mediation citrate buffer (0.01 M). Rabbit anti-mouse IL-17 (Abcam, ab91649) was used as the primary antibody, and goat anti-rabbit antibody (ZSGB-BIO) was used as the secondary one, followed by diaminobenzidine (DAB) visualization. The IL-17 positive area and density were measured by Image-Pro Plus 6.0 in three random photos for every slice.

### 2.4. Coculture of CD11c^+^ and CD4^+^ Cells

The mice were killed, and the intact tibia and femur were taken. Boons were immersed in 75% alcohol for 2-5 min. The metaphysis was cut, the bone marrow cavity was rinsed with 1 ml PBS, and flushing fluid was collected in an anticoagulating tube. After filtration (MACS SmartStrainer, 30 *μ*m), cells were centrifuged under 500 g for 10 min and then cultured in 1640 complete medium (1% penicillin streptomycin and 10% FBS) under 37°C and 5%CO_2_. GM-CSF (20 ng/ml) was added every two days. At the 7th day, CD11c^+^ cells were sorted according to the operating instructions of CD11c^+^ MicroBeads UltraPure (Miltenyi Biotec) from cultured cells. The spleen was collected and was cut up and fully grinded. After filtration, mononuclear cells were collected after a density gradient separated by Percoll. Then, CD4^+^ cells were sorted by a CD4^+^ T Cell Isolation Kit (Miltenyi Biotec). After magnetic sorting, CD11c^+^ and CD4^+^ cells (1 : 4) were cocultured with LPS (200 ng/ml) for a 3-hour culture and ATP (5 mM) was added afterwards. After a 1-hour culture, cell samples and supernatant were collected for the next flow cytometry, PCR, ELISA, and western blot experiments.

### 2.5. Culture of CD11c^+^ Cells Stimulated by IL-17

Normal Balb/c mice were killed by cervical vertebra dislocation. After being soaked in alcohol for 2-5 minutes, the femur and tibia were separated, and the attached muscle fascia was removed by sterile gauze. Then, the metaphysis was cut by aseptic ophthalmology, and the bone marrow cavity was rinsed by 1 ml PBS. After filtration with a 200 mesh, bone marrow cells were cultured in RPMI1640 complete medium with recombinant mouse granulocyte-macrophage colony-stimulating factor (rGM-CSF, 20 ng/ml). The day on which bone marrow cells were extracted was recorded as the 1st day; the culture was induced by adding fresh rGM-CSF which was added at the 3rd and 5th days. The cells were harvested on the 7th day, and CD11c^+^ cells were sorted according to the operating instructions of CD11c^+^ MicroBeads UltraPure (Miltenyi Biotec) from cultured cells. After isolation, rGM-CSF (20 ng/ml) and IL-17 (100 ng/ml) were added once; cells were collected for PCR and western blot detection after 48 h.

### 2.6. Administration of Anti-IL-1*β* in VMC Model

After the injection of the CVB3 virus (marked as the first day), the mice were injected intraperitoneally with LEAF purified anti-mouse IL-1*β* (BioLegend, 503504) and IL-1*β* isotype (BioLegend, 400916) antibody or saline from the second day, once every three days for a total of five times. Groups are listed in Table 5. On the sixteenth day, the serum, heart, and spleen of mice were taken for follow-up experiment.

### 2.7. Flow Cytometry, ELISA, and Western Blot Analysis

*Flow cytometry*: PBMCs from the spleen of mouse or cell samples from the culture system were stimulated by PMA (Multi Science, 70-CS1001)/ionomycin and BFA/monensin (Multi Science, 70-CS1002) for 4 hours. After staining with a CD4 Monoclonal Antibody- (RM4-5-) FITC (eBioscience™, 11-0042-82), fixation, and rupture of the membrane (Multi Science, 70-GAS005), IL-17A Monoclonal Antibody-PE (eBioscience™, 12-7177-81) was stained. Finally, tests were performed with Navios (Beckman).

*ELISA*: samples of serum or supernatant of cell culture system was used for the ELISA test, and tests of IL-1*β* (Andy Gene, AD3364Mo), IL-17 (Andy Gene, AD2701Mo), and IL-18 (Andy Gene, AD3376Mo) were performed according to the manufacturer of the Andy Gene ELISA kit.

*Western blot (WB)*: myocardium tissues or cell samples were lysed (Multi Science, WB020). The total protein was mixed with a SDS-PAGE buffer (Multi Science, WB004) and boiled for 10 minutes. The Bio-Rad complete set of WB reagent consumables (not listed) was used for WB experiments. The PVDF membranes were not cut. On the first day, GAPDH (D16H11) XP**®** Rabbit mAb (CST, 5174S) was incubated overnight at 4°C. On the second day, membranes were washed by TBST (0.015%) for 3 times and then incubated with another first antibody of rabbit anti-ROR gamma antibody (Abcam, ab207082), Caspase-1 (E2Z1C) rabbit mAb (CST, 24232S), IL-1 receptor rabbit mAb (Abcam, ab229051), or NLRP3 (D4D8T) rabbit mAb (CST, 15101S) at 37°C for 2 hours; after another 3-times wash of TBST, the second antibody, anti-rabbit IgG, HRP-linked antibody (CST, 7074S), was incubated at 37°C for 1 hour. The exposure uses a Bio-Rad ChemiDoc™ Imaging System, fluorescence grayscale pictures were taken under nonlight dark field, and original pictures of PVDF membranes were taken subsequently under white light and then merged for the synthetic picture with protein marker. Bio-Rad Image Lab 6.0 was used to analyze the grayscale pictures. The expression value was calculated by protein expression gray value (Int)/internal reference GAPDH gray value (Int). The protein marker was from Bio-Rad (161-0393). The kd values of each band are marked on the graph.

### 2.8. qPCR and Relative Quantification

The total RNA of cells or tissues was extracted by an RNAiso Plus reagent (TaKaRa). PrimeScript™ RT Reagent Kit with gDNA Eraser (TaKaRa) was used for reverse transcription. SYBR® Premix Ex Taq™ II (TaKaRa) was used in real-time PCR reaction (7500 Real-Time PCR Instrument, Life). The entire process was carried out according to the manufacturer's instructions. The 20 *μ*l PCR reaction contained SYBR green, ROX, primers, cDNA, and RNase-free water. The relative quantification was calculated by -2^*ΔΔ*CT^or -2^*Δ*CT^. Each analysis was repeated three times.

### 2.9. Statistics

Spearman was used to analyze the correlation between NLRP3 and Th17. Statistical testing was performed by Student's *t* test, one-way ANOVA, or two-way ANOVA test with multiple comparisons by GraphPad Prism 7 unless otherwise indicated. Statistical significance was assumed at *P* < 0.05.

## 3. Results

### 3.1. Establishment of an Ideal Viral Myocarditis Model in Balb/c Mice

**Figure 1:**
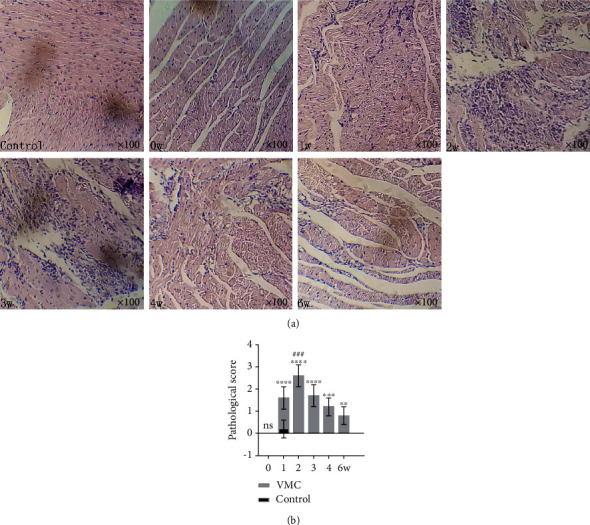
Myocardial pathological changes in VMC process. (a) HE images of inflammation during the six-week VMC modeling process (original magnification ×100). (b) Pathological score at different time points. Statistical description: ns, ∗∗, ∗∗∗, and ∗∗∗∗ stand for no statistically significant difference, *P* < 0.01, *P* < 0.001, and *P* < 0.0001, respectively, for the VMC group versus the control group at different time points. *###* means *P* < 0.001 for the week 2 VMC group versus week 0, 1, 3, 4, and 6 VMC groups.

**Figure 2:**
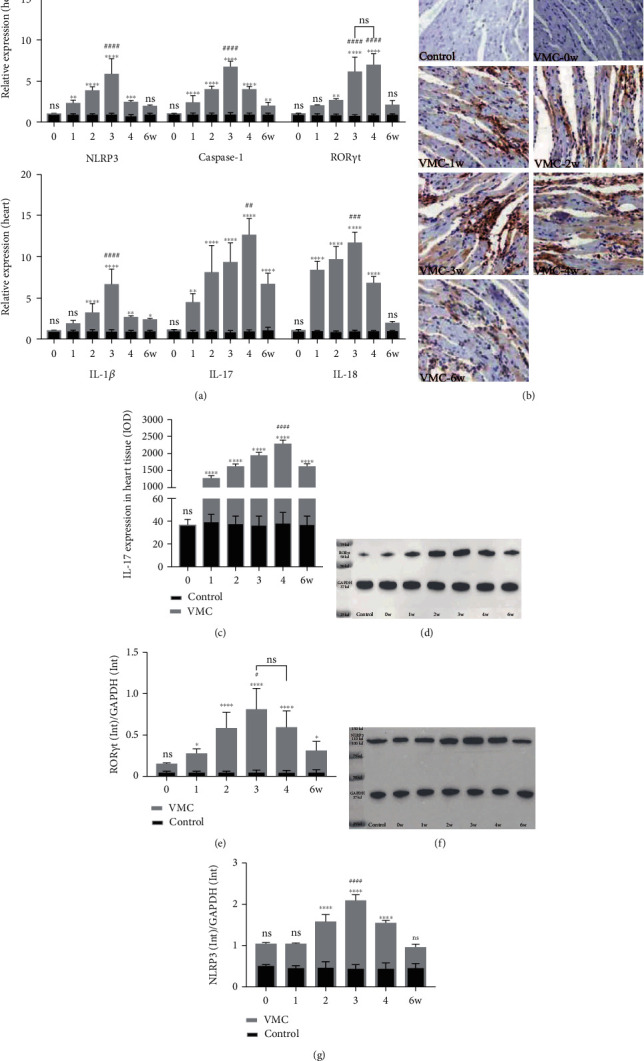
The change pattern of NLRP3-related molecules in myocardial tissue in VMC process. (a) Relative mRNA expression of NLRP3, Caspase-1, ROR*γ*t, IL-1*β*, IL-17, and IL-18 in heart tissues in CVB3-induced VMC. (b) IL-17 expression in heart tissue detected by IHC (original magnification ×400). (c) IL-17 expression in heart tissue acquired by IHC assay, and IOD value was calculated by Image-Pro Plus. (d, e) The protein expression of ROR*γ*t in heart tissue was detected by WB and calculated by Bio-Rad Image Lab 6.0. (f, g) The protein expression of NLRP3 in heart tissue was detected by WB and calculated by Bio-Rad Image Lab 6.0. Statistical description: ns, ∗, ∗∗, ∗∗∗, and ∗∗∗∗ stand for no statistically significant difference, *P* < 0.05, *P* < 0.01, *P* < 0.001, and *P* < 0.0001, respectively, for VMC group versus control group at different time points. ## and ### mean *P* < 0.001 for the marked VMC group versus other VMC groups.

**Figure 3:**
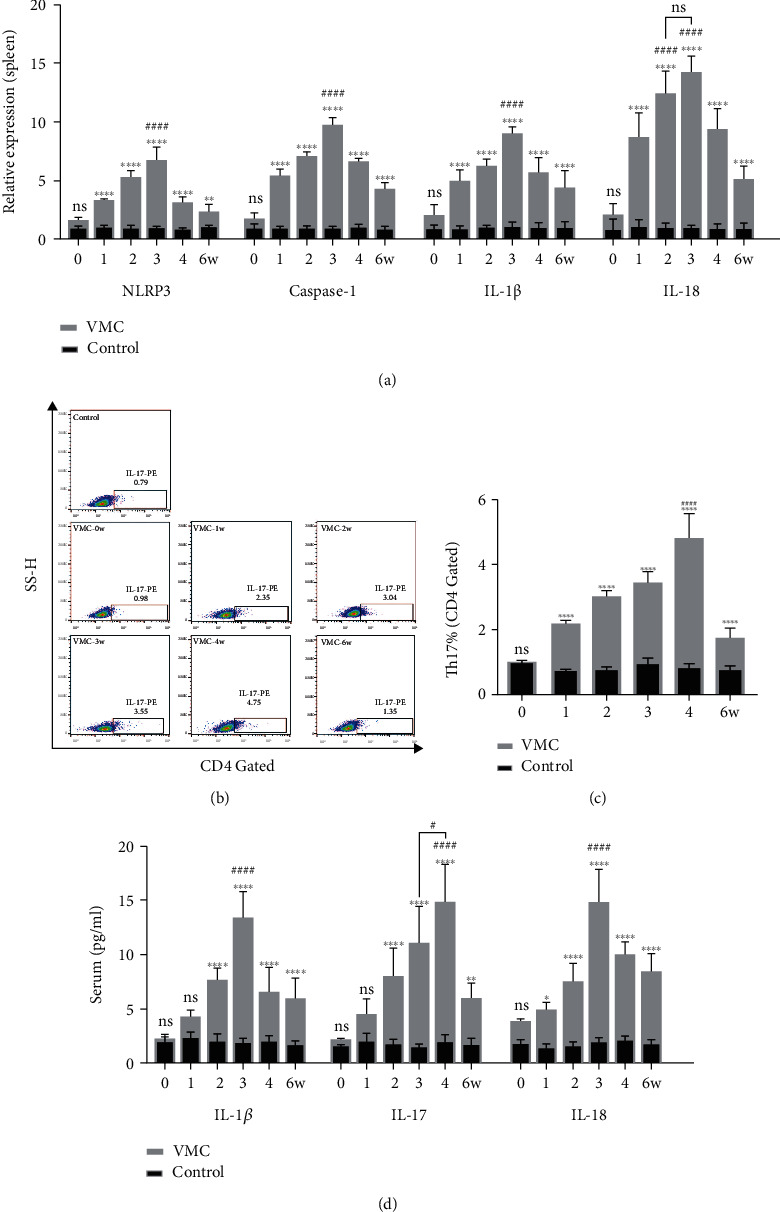
The change pattern of NLRP3-related molecules in the spleen and serum in VMC process. (a) Relative mRNA expression of NLRP3, Caspase-1, IL-1*β*, and IL-18 in spleen tissues during VMC process. (b) The proportion of Th17 cells in spleen samples during VMC process. (c) Statistical histogram of Th17 cell proportion calculation. (d) Protein level of IL-1*β*, IL-17, and IL-18 in serum during VMC process. Statistical description: ns, ∗, ∗∗, and ∗∗∗∗ stand for no statistically significant difference, *P* < 0.05, *P* < 0.01, and *P* < 0.0001, respectively, for VMC group versus control group at different time points. #, ###, and #### mean *P* < 0.05, *P* < 0.001, and *P* < 0.0001 for the marked VMC group versus other VMC groups.

**Figure 4:**
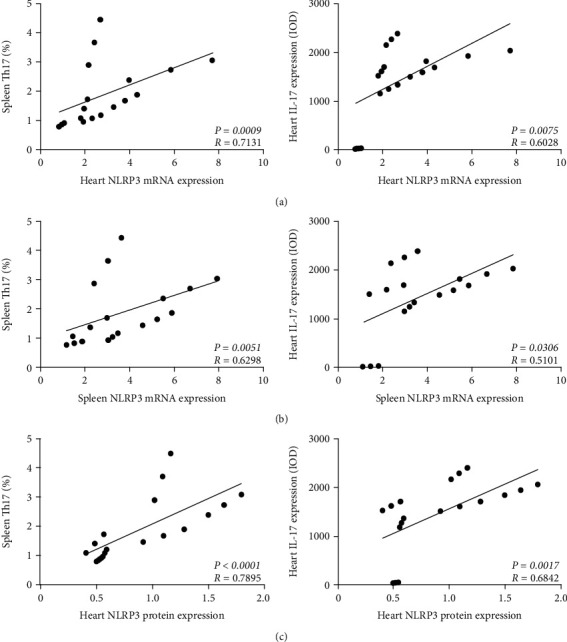
Correlation analysis of NLRP3 inflammasome and Th17 cells. (a) Correlation analysis between NLRP3 (mRNA level in the heart) and Th17 cells (proportion in the spleen, left) or IL-17 expression (IOD score detected by IHC in heart, right). (b) Correlation analysis between NLRP3 (mRNA level in the spleen) and Th17 cells (proportion in the spleen, left) or IL-17 expression (IOD score detected by IHC in the heart, right). (c) Correlation analysis between NLRP3 (protein level in the heart) and Th17 cells (proportion in the spleen, left) or IL-17 expression (IOD score detected by IHC in the heart, right). *P*: *P* value; *R*: Spearman *R*.

The mice in the VMC group showed symptoms such as shrugging, tiredness, hunchback, dull fur, reduced activity, insensitivity to stimulation, or irritability since the third day after infection with CVB3, while there was no abnormal appearance in the control group. The results of histopathological examination showed that at the initial stage of the VMC model, the myocardial tissue was mainly characterized by cell swelling, focal necrosis, and a small amount of inflammatory cell infiltration (Figure 1(a)-1w). Then, it developed to the peak of inflammation, which was mainly characterized by multispot or even patchy necrosis (Figure 1(a)-2w and 3w). Then, the infiltration of inflammatory cells decreased gradually, and fibrous formation could be seen (Figure 1(a)-4w and 6w). The pathological score showed that the inflammation of the VMC model reached its peak at the second week, which was significantly different from that at other time points (Figure 1(b)).

### 3.2. The Regularity of NLRP3 and Th17 Cells in the Pathological Process of CVB3-Induced VMC

In order to explore the change pattern of NLRP3 and its associated cytokines in CVB3-induced VMC, CVB3 was intraperitoneally injected into six groups (0, 1, 2, 3, 4, and 6w) of Balb/c mice. Spleen, heart, and peripheral blood samples were collected from the corresponding group every week until the sixth week. In the heart tissues, the mRNA level of NLRP3 raised at the first week and peaked at the third week. After that, it gradually decreased to the level close to that of the control group. Besides, the mRNA level of Caspase-1, IL-1*β*, and IL-18 and those downstream molecules of NLRP3 showed a similar changing pattern in the heart tissues during the process of CVB3-induced VMC (Figure 2(a)). The mRNA level of ROR*γ*t in the heart tissues, a marker of Th17 cells, which can promote thymocyte differentiation into proinflammatory Th17 cells [32, 33], peaked at the third and fourth weeks (Figure 2(a)). Then, we measured the expression of IL-17 in heart tissues by IHC. As shown in Figures 2(b) and 2(c), the expression of IL-17 peaked at the fourth week in the heart tissue of VMC mice. Western blot was used to further confirm the change pattern of ROR*γ*t and NLRP3 at the protein level. As shown in Figures 2(d)–2(g), the protein levels of ROR*γ*t and NLRP3 both peaked at the third week and were followed by a gradual decrease. Similarly, the mRNA level of NLRP3, Caspase-1, IL-1*β*, and IL-18 showed the same pattern of change in the spleen tissues during the process of CVB3-induced VMC (Figure 3(a)). Then, the proportion of Th17 cells in spleen samples was analyzed by flow cytometry. In accordance with the expression of IL-17 in the heart tissue, the proportion of Th17 in the spleen also peaked at the fourth week (Figures 3(b) and 3(c)). Finally, the concentrations of IL-1*β*, IL-17, and IL-18 in serum samples were analyzed by ELISA. As shown in Figure 3(d), the protein levels of IL-1*β* and IL-18 in the serum peaked at the third week, while the concentration of IL-17 peaked at the fourth week.

### 3.3. NLRP3 Is Significantly Positively Correlated to Th17 in the Pathological Process of CVB3-Induced VMC

After collecting PCR, WB, and flow cytometry data, we analyzed the correlation between NLRP3 and Th17 cells. After Spearman correlation coefficient analysis, as shown in Figure 4, the mRNA or protein levels of NLRP3 in the myocardium were positively correlated with Th17 cells in the spleen (Figure 4(a), left and Figure 4(c), left). The mRNA level of spleen NLRP3 was also significantly correlated with spleen Th17 cells (Figure 4(b), left), but the coefficient was lower than that between myocardial NLRP3 expression and spleen Th17 expression. In addition, we also analyzed the correlation between the expression of NLRP3 and myocardial IL-17 and found that the mRNA level of NLRP3 in the myocardium or spleen was also positively correlated with the expression of myocardial IL-17, and the correlation between the mRNA levels of spleen NLRP3 and myocardial IL-17 was lower (Figure 4(a), right, and Figure 4(b), right). Similarly, the protein level of NLRP3 in the myocardium was significantly positively correlated with the expression of IL-17 in the myocardium (Figure 4(c), right).

### 3.4. Orchestration of NLRP3 Inflammasome and Th17 Cells in CVB3-Induced Myocarditis

**Figure 5:**
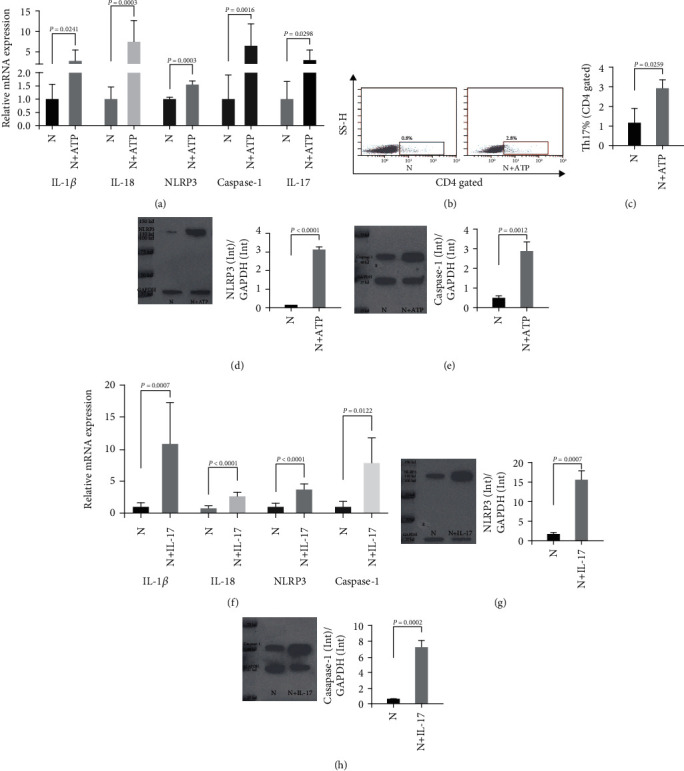
Orchestration of NLRP3 inflammasome and Th17 cells in CVB3-induced myocarditis. (a) The relative mRNA expression of IL-1*β*, IL-18, NLRP3, Caspase-1, and IL-17 in N and N+ATP groups in the coculture system. (b) The proportion of Th17 cells in N and N+ATP groups in the coculture system. (c) Statistic calculation column of Th17 proportion in N and N+ATP groups in the coculture system. (d) The protein level of NLRP3 in N and N+ATP groups in the coculture system. (e) The protein level of Caspase-1 in N and N+ATP groups in the coculture system. (f) The relative mRNA levels of IL-1*β*, IL-18, NLRP3, and Caspase-1 in the CD11c^+^ DC cells stimulated with or without IL-17. (g) The protein level of NLRP3 in CD11c^+^ DC cells stimulated with or without IL-17. (h) The protein level of Caspase-1 in CD11c^+^ DC cells stimulated with or without IL-17. (a–e) were acquired from the CD11c^+^ DC and CD4^+^ T cell coculture system stimulated by LPS with (N+ATP) or without ATP (N). (f–h) were acquired from CD11c^+^ DC cells stimulated with IL-17 (N+IL-17) or without IL-17 (N). Statistical description: the *P* value in the figure has been marked between the corresponding groups.

A coculture system was set up to explore the influence of NLRP3 on Th17 cells. CD4^+^ T and CD11c^+^ DC cells were sorted from the spleen and bone marrow, respectively, in normal Balb/c. First, the mRNA levels of IL-1*β*, IL-18, IL-17, NLRP3, and Caspase-1 and those NLRP3-associated molecules were checked in the cell samples in the coculture system. In the coculture system, the mRNA expression of IL-1*β* (*P* = 0.0241), IL-18 (*P* = 0.0003), NLRP3 (*P* = 0.0003), Caspase-1 (*P* = 0.0016), and IL-17 (*P* = 0.0298) was significantly higher after being cultured with ATP (N vs. N+ATP) (Figure 5(a)). Then, the proportion of Th17 cells in the coculture system was detected. The proportion of Th17 in the N+ATP group was significantly higher than that in the N group (*P* = 0.0259, N vs. N+ATP) (Figures 5(b) and 5(c)). Finally, the protein levels of NLRP3 and Caspase-1 were detected by WB. The protein level of NLRP3 in the N+ATP group was significantly higher than that in the N group (Figure 5(d), *P* < 0.0001, N vs. N+ATP). The protein level of Caspase-1 in the N+ATP group was also significantly higher than that in the N group (Figure 5(e), *P* < 0.0012, N vs. N+ATP). In addition, no sufficient statistical differences were observed in the expression of IL-1*β*, IL-18, and IL-17 in the supernatant of each group.

In turn, when studying the effect of Th17 on the NLRP3 inflammasome, the research group selected IL-17, the main proinflammatory cytokine of Th17, to stimulate CD11c^+^ cells cultured in vitro to study its possible effect on NLRP3. First, we explored the changes of the mRNA level of NLRP3 itself and its downstream factors with or without IL-17 stimulation (N vs. N+IL-17). It was found that IL-17 could significantly upregulate the mRNA expression of IL-1*β*, IL-18, NLRP3, and Caspase-1 (Figure 5(f)). Then, the protein levels of NLRP3 and Caspase-1 were compared between the N and N+IL-17 groups. It showed that the protein expression of both NLRP3 (Figure 5(g)) and Caspase-1 (Figure 5(h)) increased significantly under the stimulation of IL-17, which was in accordance with the mRNA expression. Of course, the research group also detected IL-1*β* and IL-18 in the supernatant. Unfortunately, no significant difference was found between the groups with or without IL-17.

### 3.5. Blockade of IL-1*β* Pathway Significantly Alleviates the Severity of CVB3-Induced VMC in Balb/c

**Figure 6:**
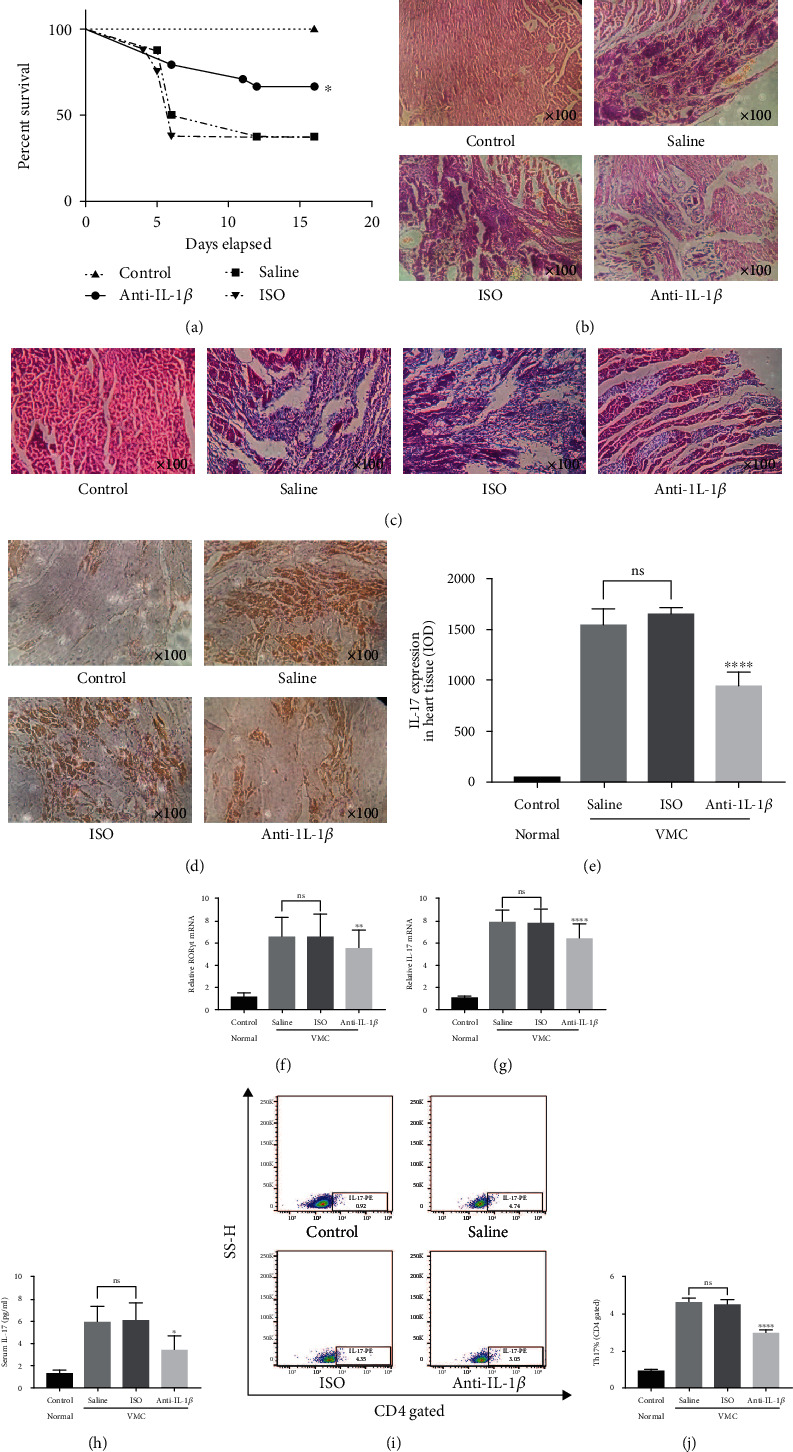
Blockade of IL-1*β* can significantly alleviate the severity of VMC in Balb/c. (a) Statistical analysis of survival rate from day zero to day sixteen in control, anti-IL-1*β*, saline, and ISO groups. (b) HE images of myocardial in control, anti-IL-1*β*, saline, and ISO groups at sixteenth day of CVB3-induced VMC process (original magnification ×100). (c) Masson-trichrome staining of myocardial in control, saline, ISO, and anti-IL-1*β* groups at sixteenth day of CVB3-induced VMC process (original magnification ×100). (d) IHC images of IL-17 in myocardial in control, saline, ISO, and anti-IL-1*β* groups at sixteenth day of CVB3-induced VMC process (original magnification ×100). (e) IL-17 expression in heart tissue acquired by IHC assay and IOD value was calculated by Image-Pro Plus. (f, g) The relative mRNA expression of ROR*γ*t and IL-17 in myocardium in control, anti-IL-1*β*, saline, and ISO groups at sixteenth day of CVB3-induced VMC process. (h) The protein level of IL-17 in plasma in control, anti-IL-1*β*, saline, and ISO groups at sixteenth day of CVB3-induced VMC process. (i) The proportion of Th17 cells in spleen samples in control, anti-IL-1*β*, saline, and ISO groups at sixteenth day of CVB3-induced VMC process. (j) Statistical histogram of Th17 proportion in (i). Statistical description: ns, ∗, ∗∗, and ∗∗∗∗ stand for no statistically significant difference, *P* < 0.05, *P* < 0.01, and *P* < 0.0001, respectively, for anti-IL-1*β* group versus saline or ISO group.

The symptoms of shrugging, shrinkage, hunchback, dull fur, decreased activity, insensitivity to stimulation, or irritability in saline and ISO groups began to appear on the third day after injection of CVB3. Mice in the anti-IL-1*β* group were slightly better; their hair shrug and curl up were lighter, and their free movement and eating ability were slightly stronger than those in the ISO and saline groups. There were eight mice in each group. After three independent experiments, a total of twenty-four mice in each group were used to calculate the survival rate (Figure 6(a)). The survival rate of the anti-IL-1*β* group was significantly higher than those of the saline group and the ISO group (IL-1*β* vs. saline, *P* = 0.032; IL-1*β* vs. saline, *P* = 0.015).

Next, HE sections were used to explore the pathological status of myocarditis in each group. The control group was a negative control of normal mice with obvious myocardial texture and no inflammatory infiltration. Mice in the saline and ISO groups showed a large number of multifocal necrosis and inflammatory cell infiltration, and the original structure of the myocardium was destroyed. However, there was no large amount of inflammatory exudation in HE sections of the myocardium in the anti-IL-1*β* group, and the situation of focal myocardial necrosis in the anti-IL-1*β* group was significantly better than that in the saline and ISO groups (Figure 6(b)). Besides, Masson-trichrome staining (Figure 6(c)) showed less pronounced fibrosis in the anti-IL-1*β*-treated mice than in the ISO- and saline-treated mice.

On the other hand, the contents of Th17 and IL-17, which are closely related to the occurrence and development of VMC [7], were also detected. In the IHC test, a large number of brown IL-17-positive areas were found in the saline and ISO groups. However, the content of IL-17 in the anti-IL-1*β* group was significantly lower than that in the saline and ISO groups (Figures 6(d) and 6(e)). The relative mRNA levels of IL-17 and ROR*γ*t mRNA in the myocardium were measured by qPCR. As shown in Figures 6(f) and 6(g), the mRNA levels of ROR*γ*t and IL-17 in the anti-IL-1*β* group were significantly lower than those in the saline and ISO groups. Similarly, the content of IL-17 in the serum (Figure 6(h)) and the proportion of Th17 cells in the spleen (Figures 6(i) and 6(j)) in the anti-IL-1*β* group were also significantly lower than those in the saline and ISO groups.

### 3.6. Knockout of NLRP3 Significantly Alleviates the Severity of CVB3-Induced VMC in Balb/c

**Figure 7:**
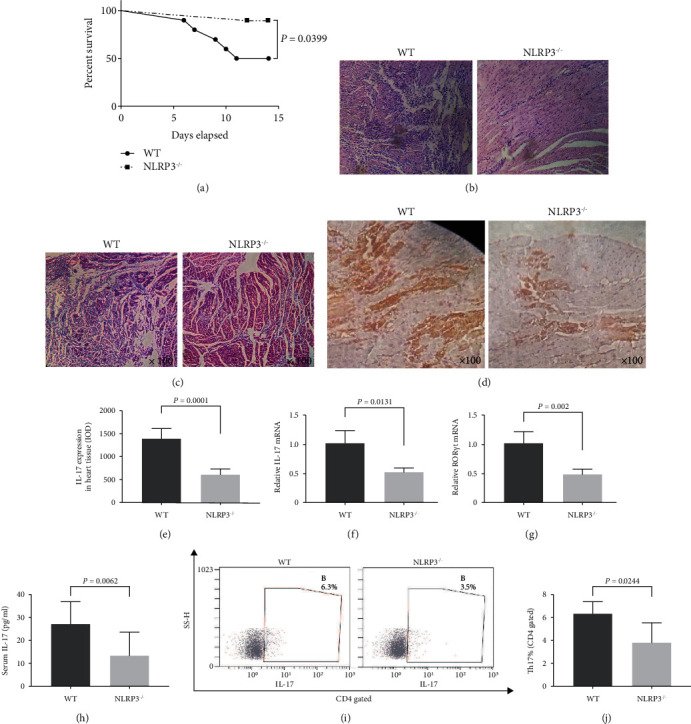
Knockout of NLRP3 significantly alleviates the severity of CVB3-induced VMC in Balb/c. (a) Statistical analysis of survival rate from day zero to day fourteen in WT and NLRP3^−/−^ group. (b) HE images of myocardial in WT and NLRP3^−/−^ groups at fourteenth day of CVB3-induced VMC process (original magnification ×100 and ×200). (c) Masson-trichrome staining of myocardial in WT and NLRP3^−/−^ groups at fourteenth day of CVB3-induced VMC process (original magnification ×100). (d) IHC images of IL-17 in myocardial in WT and NLRP3^−/−^ groups at fourteenth day of CVB3-induced VMC process (original magnification ×100). (e) IL-17 expression in heart tissue acquired by IHC assay and IOD value was calculated by Image-Pro Plus. (f, g) The relative mRNA expression of IL-17 and ROR*γ*t in myocardium in WT and NLRP3^−/−^ groups at fourteenth day of CVB3-induced VMC process. (h) The protein level of IL-17 in plasma in WT and NLRP3^−/−^ groups at fourteenth day of CVB3-induced VMC process. (i) The proportion of Th17 cells in spleen samples in WT and NLRP3^−/−^ groups at fourteenth day of CVB3-induced VMC process. (j) Statistical histogram of Th17 proportion in (i). Statistical description: the *P* value in the figure has been marked between the corresponding groups.

Under the intervention of the NLRP3 knockout, the mortality of VMC caused by CVB3 was significantly reduced (Figure 7(a)). In the HE staining assay, obvious inflammatory infiltration was observed in the myocardium in the WT group, which destroyed the original myocardial structure and appeared the pathological phenomenon of multifocal necrosis such as cardiomyolysis. In the NLRP3^−/−^ group, there was only mild inflammatory infiltration, the myocardial structure was still intact, and there was no obvious necrosis (Figure 7(b)). Masson-trichrome staining (Figure 7(c)) showed less pronounced fibrosis in the NLRP3^−/−^ mice than in the WT mice. As shown in Figures 7(d) and 7(e), the expression of IL-17 in the myocardium in the NLRP3^−/−^ group was significantly lower than that in the WT group detected by IHC. On the other hand, the mRNA levels of IL-17 (Figure 7(f)) and ROR*γ*t (Figure 7(g)) in the myocardium in the NLRP3^−/−^ group were significantly lower than those in the WT group. In addition, the content of plasma IL-17 in the NLRP3^−/−^ group was significantly lower than that in the WT group (Figure 7(h)). Finally, we measured the proportion of Th17 cells in the spleen in each group at the second week after CVB3 infection. Similarly, the proportion of Th17 in the spleen in the NLRP3^−/−^ group was significantly lower than that in the WT group (Figures 7(i) and 7(j)).

## 4. Discussion

The NLRP3 inflammasome plays an important role in the pathogenesis of some cardiovascular diseases, such as myocardial ischemia/reperfusion injury [34], acute myocardial infarction [35], and ventricular remodeling after myocardial infarction [36]. Recent studies have shown that the NLRP3 inflammasome is also involved in the pathological process of CVB3-induced myocarditis, which describes an upregulation of NLRP3 triggered by CVB3 capsid proteins VP1 and VP2, and an activation of NLRP3 mediated by ROS production and K+ efflux [5, 37]. However, it is still unknown whether the overactivated NLRP3 is involved in the whole pathological process of VMC and how it changes. After analysis of a large amount of data, for the first time, the change patterns of NLRP3-related and Th17-related factors in the myocardial tissue, spleen, and peripheral blood were illustrated at the mRNA level, protein level, and cell level in the whole process of pathological changes of the viral myocarditis model induced by CVB3. In the aggravating stage of VMC after CVB3 injection, the expression of NLRP3 in the myocardium increased gradually at both the mRNA level and the protein level. At about the second to the third week, when the pathological manifestation of myocarditis was the most severe, the expression of NLRP3 reached the peak. Then, with the gradual relaxation of VMC, the expression of NLRP3 decreased, suggesting that NLRP3 is closely related to the pathological condition of CVB3-induced VMC. In addition, in the study, we further found that there was a similar changing pattern of Th17 cell proportion and its related factors IL-17 and ROR*γ*t.

Th17 cells are a subset of T helper cells related to autoimmunity and inflammation, which have IL-1*β* receptors and can secrete cytokines such as IL-17 and IL-22 under the stimulation of IL-1*β* [38]. While NLRP3 is the component that processes IL-1*β* into a mature cytokine [39], the activation of the NLRP3 inflammasome can promote the production of IL-1*β* and IL-18. Besides, Yang et al. [7] indicated that the IL-23/Th17 pathway is strongly expressed in murine VMC. All of these suggest that there may be some relationship between NLRP3 and Th17 cells in the occurrence and development of VMC. Therefore, Spearman analysis was used to explore the correlation between NLRP3 inflammasome and Th17 in the whole pathological process of VMC. Surprisingly, the results showed that NLRP3 showed a significant correlation with Th17 or its main effector IL-17, whether at the mRNA level or at the protein level.

However, it is still unknown whether or what kind of interaction between the NLRP3 inflammasome and Th17 exists in the process of CVB3-induced VMC. Thus, two more vitro experiments were designed.

When exploring the effect of NLRP3 on Th17, spleen CD4^+^ T cells and bone marrow CD11c^+^ cells from the normal Balb/c mouse were cocultured in vitro. LPS was supplied to prime the NLRP3 inflammasome [4]. Then ATP, an agonist of NLRP3 [40, 41], was used to activate the NLRP3 pathway to observe the response of cocultured cells. In light of the expression of Caspase-1 and NLRP3 proteins, the addition of ATP can significantly increase the expression of these two proteins, which is consistent with the literature that ATP is used as a NLRP3 agonist. Then, IL-1*β*, IL-18, Caspase-1, and these downstream cytokines of NLRP3 can respond quickly under the activation of NLRP3 induced by ATP and show increased mRNA expression. In addition, the mRNA expression of IL-17 and the proportion of Th17 cells in the N+ATP group was significantly higher than that in the N group, suggesting that the priming of LPS and the activation of NLRP3 by ATP could promote CD4^+^ T cells differentiating into Th17 cells, and induced a positive regulation of Th17 cells in the CD4^+^ T cells and CD11c^+^ DC coculture system.

In turn, when exploring the effect of Th17 on the NLRP3 inflammasome, the research group selected IL-17, the main proinflammatory cytokine of Th17 [42], to study its possible effect on NLRP3. The magnetic sorting CD11c^+^ DC cells derived from the bone marrow of Balb/c mice were cultured with or without IL-17 during routine GM-CSF stimulation. In the analysis of PCR results in each group, we found that the addition of IL-17 could significantly upregulate the mRNA levels of IL-1*β*, IL-18, NLRP3, and Caspase-1.

Therefore, an orchestration of the NLRP3 inflammasome and Th17 cells in CVB3-induced myocarditis is confirmed. Next, we move on to explore whether this interaction between NLRP3 and Th17 can be used in the therapy strategy of CVB3-induced VMC. Pathological analysis and Th17-related factors were selected as the prognostic indexes of CVB3-induced VMC under the intervention of the anti-IL-1*β* antibody and NLRP3 knockout.

The IL-1*β* pathway was blocked by intraperitoneal injection of the anti-IL-1*β* antibody, and the pathological changes of VMC were compared with those in the control, ISO, and saline groups. It was confirmed that the IL-1*β* pathway blockage could reduce the mortality of VMC mice, significantly alleviate the myocardial inflammation and fibrosis caused by VMC process, reduce the exudation of myocardial inflammatory cells, and reduce the expression of IL-17 protein and mRNA in the myocardium during the pathological process of VMC. The expression of IL-17 in the peripheral blood and the proportion of Th17 cells in the spleen were also significantly downregulated using the IL-1*β* antibody. This part of the results suggests that in the VMC model of Balb/c mice, the blockage of the IL-1*β* pathway, downstream of the NLRP3 inflammasome, can significantly alleviate the severity of VMC in mice, but still cannot achieve the purpose of complete remission. Considering that the anti-IL-1*β* antibody can only partially block the NLRP3 pathway, NLRP3 knockout is used as a complete blockage of the NLRP3 pathway. Under this intervention, the effect of the NLRP3 pathway on CVB3-induced VMC was reanalyzed. Results showed that NLRP3 knockout could significantly reduce the mortality of VMC caused by CVB3, and the degree of myocardial inflammation or fibrosis was mild, mainly characterized by less inflammatory cell infiltration and less cardiomyocyte injury. In addition, the results also indicated that the expression of IL-17 in the myocardium was significantly lower in the NLRP3 knockout group, and the proportion of Th17 cells in spleen mononuclear cells was also significantly lower when compared with that in the WT group. Furthermore, in order to control the variables, we also compared the expression of the IL-1*β* receptor in the heart and spleen tissues between WT and NLRP3^−/−^ mice, which shows no statistical difference (Figure S1).

Collectively, a certain relationship can be determined between the NLRP3 inflammasome and Th17 during the pathological process of CVB3-induced VMC, when both CD11c^+^ DC and CD4^+^ T cells in the coculture system were from normal Balb/c mice. The increased expression of NLRP3 can promote the differentiation of CD4 cells into Th17 cells, and the IL-17 expressed by Th17 cells can in turn promote the expression of NLRP3, which suggests a positive feedback regulatory mechanism between NLPR3 and Th17 cells in vitro and in the early progressive stage of CVB3 infection. However, when CD11c^+^ DC or CD4^+^ T cells in the coculture system were from Balb/c mice enduring the peak of inflammation or the subsequent recovery period, the experimental results showed great uncertainty, and the repeatability between independent experiments was very poor. Thus, the positive feedback loop between the NLRP3 inflammasome and Th17 cannot be deduced in the peak and recovery period of myocarditis. Of course, this complex mechanism is more interesting and worth studying and exploring, but considering the great difference in the characteristics of immune cells from the immune initiation of viral infection to the possible stage of immune exhaustion, the study is extremely difficult. Therefore, only part of the results can be published in advance. Further research is also ongoing. On the other hand, the experiment in anti-IL-1*β* and NLRP3^−/−^ knockout sections fully confirmed that in Balb/c mice, the disruption or block of the NLRP3 pathway could significantly improve the prognosis of VMC caused by CVB3 and greatly alleviate the pathological process of VMC. Putting forward a new idea of VMC treatment by means of NLRP3 blocking, further clinical trials are urgently needed.

## Figures and Tables

**Table 1:** The sequence and concentration of gRNA used in NLRP3 KO.

gRNA	gRNA sequence (5′ to 3′)	gRNA (con.)
Nlrp3-gRNA1	AGGTGGTATGACCGGACAGAGGG	4750 ng/*μ*l
Nlrp3-gRNA3	ATTCCGTCGTTCTGAGACGATGG	4950 ng/*μ*l

**Table 2:** Primers used in NLRP3 identification.

Primer	Sequence (5′ to 3′)	
Nlrp3-seq-F1	AGTGCTGGCTTTCTGCCCTATCA	KO
Nlrp3-seq-F2	GCTGTGATGGATGCCAGATTGAAG	WT
Nlrp3-seq-R	GGTCCTTGTCAGGCTGTTGATTGTT	General

**Table 3:** Criteria used in genotype determination.

	F1+R	F2+R
WT (+/+)	No product	0.37 kb
Heter (+/-)	0.5 kb	0.37 kb
Homo (-/-)	0.5 kb	No product

**Table 4:** Pathology semiquantitative scale.

Score	Pathological description
0	No inflammatory infiltrates
1	Small foci of inflammatory cells between myocytes or inflammatory cells surrounding individual myocytes
2	Larger foci of 100 inflammatory cells or involving at least 30 myocytes
3	10% of a myocardial cross-section involved
4	30% of a myocardial cross-section involved

**Table 5:** Groups of VMC mice treated with anti-IL-1*β* antibody.

Groups	CVB3	Anti-IL-1*β* (I.P. 100 *μ*g/time)	Saline (equal volume)	ISO (I.P. 100 *μ*g/time)
Control	−	−	+	−
Anti-IL-1*β*	+	+	+	−
Saline	+	−	+	−
ISO	+	−	+	+

## Data Availability

The data generated or analyzed during this study are included in the main text and its additional files. Further details in our study are also available upon reasonable request.

## Abbreviations

NLRP3: NOD-, LRR-, and pyrin domain-containing protein 3
VMC: Viral myocarditis
CVB3: Coxsackie virus group B type3
NOD2: Nucleotide-binding oligomerization domain-containing protein 2
PAMP: Pathogen-associated molecular pattern
PRR: Pattern recognition receptor
TLRs: Toll-like receptors
NLRs: Nucleotide-binding oligomerization domain-like receptors
ROS: Reactive oxygen species.

## Additional Points

*Code Availability*. The subject does not involve the analysis, processing, and application of related codes and data.

## Supplementary Materials

Supplementary 1 FIG S1: the protein level of IL-1R in myocardial and spleen tissues in WT and NLRP3-/- mice.

Supplementary 2 Review report of the ethics committee for the “orchestration of NLRP3 inflammasome and Th17 cells in CVB3-induced myocarditis.”

Supplementary 3 Review report of the ethics committee for the “blockade of the NLRP3 inflammasome improves the prognosis of CVB3-induced myocarditis in Balbc mice.”
