# Supplementary material for: The Severity of CVB3-Induced Myocarditis Can Be Improved by Blocking the Orchestration of NLRP3 and Th17 in Balb/c Mice
Source: Mediators Inflamm. 2021 May 12;2021:5551578. doi: 10.1155/2021/5551578 (PMC8139334; doi:10.1155/2021/5551578)
Supplement: Supplementary 1 — FIG S1: the protein level of IL-1R in myocardial and spleen tissues in WT and NLRP3-/- mice. [file 5551578.f1.zip › Figure S1.docx]

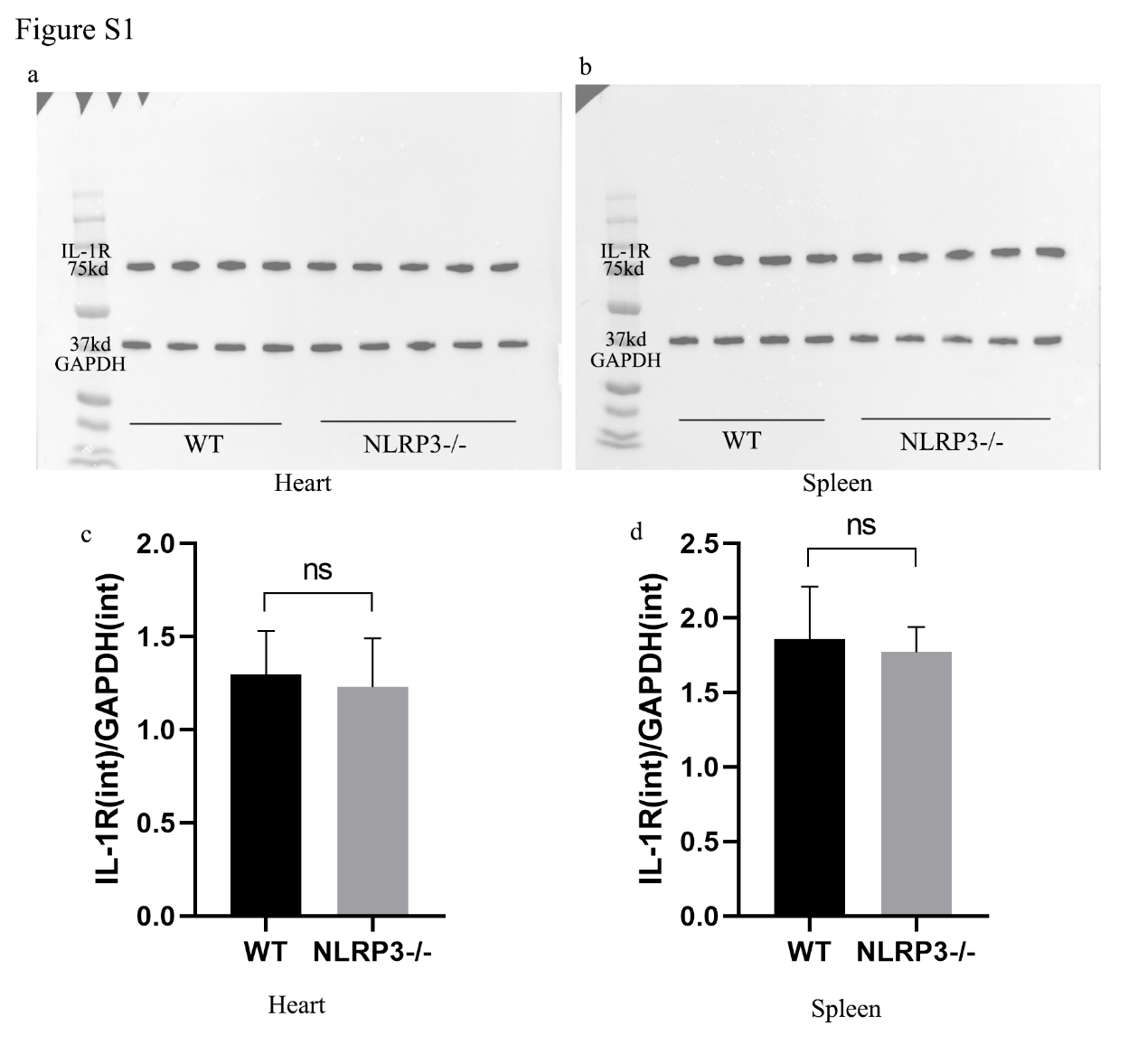


**Figure S1. The protein level of IL-1R in myocardial and spleen tissues in WT and NLRP3^-/-^ mice**

Figure legend of figure S1. a and c. The protein level of IL-1R in myocardial tissues in WT and NLRP3^-/-^ mice WT and and calculated column figure. b and d. The protein level of IL-1R in spleen tissues in WT and NLRP3^-/-^ mice and calculated column figure.
