# Supplementary figures and images for: The Severity of CVB3-Induced Myocarditis Can Be Improved by Blocking the Orchestration of NLRP3 and Th17 in Balb/c Mice
Source: Mediators Inflamm. 2021 May 12;2021:5551578. doi: 10.1155/2021/5551578 (PMC8139334; doi:10.1155/2021/5551578)

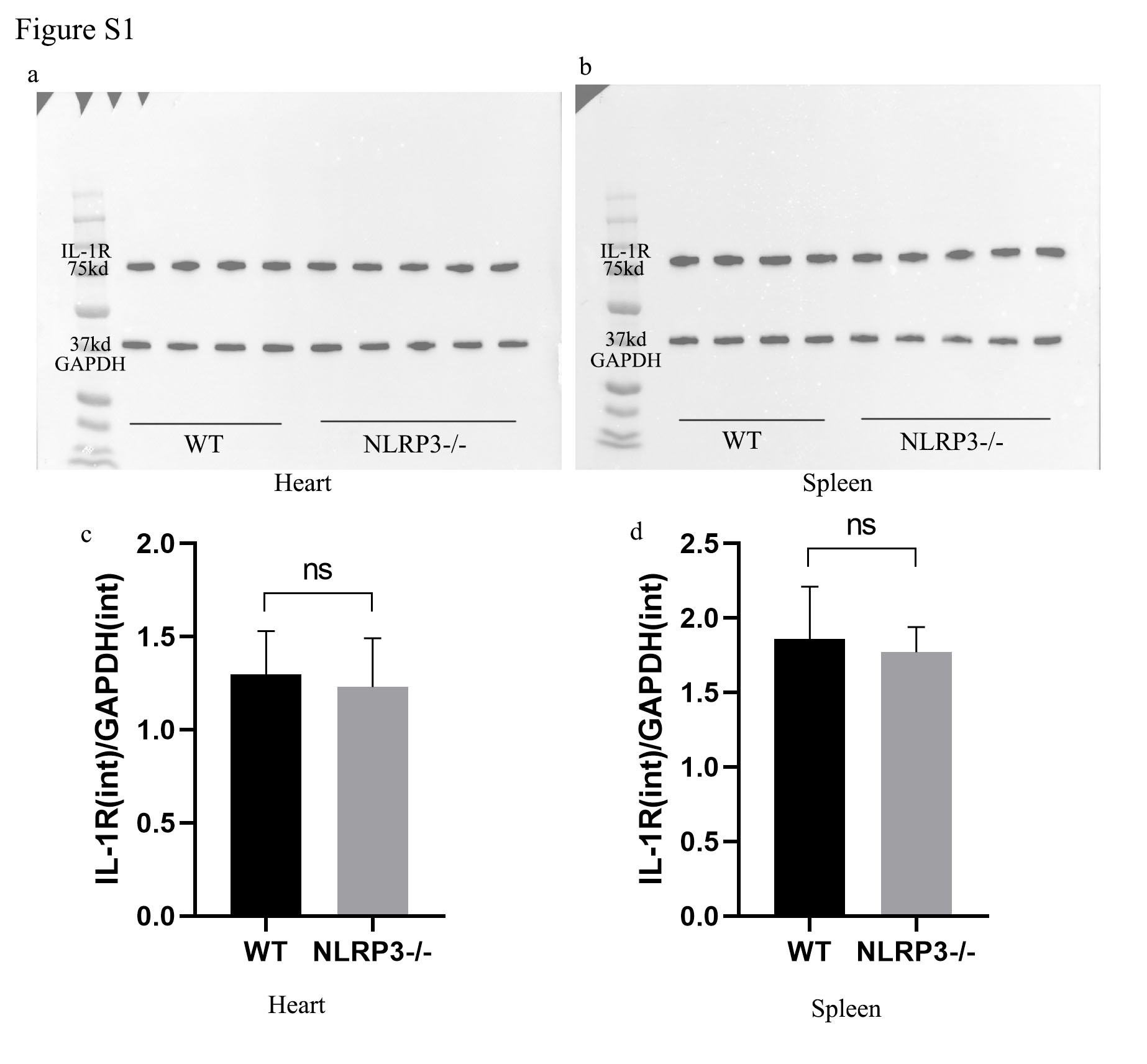

Supplement: Supplementary 1 — FIG S1: the protein level of IL-1R in myocardial and spleen tissues in WT and NLRP3-/- mice. [file 5551578.f1.zip › FIG S1.jpg]
